# Epidemiology and Burden of Human Papillomavirus and Related Diseases, Molecular Pathogenesis, and Vaccine Evaluation

**DOI:** 10.3389/fpubh.2020.552028

**Published:** 2021-01-20

**Authors:** Arnaud John Kombe Kombe, Bofeng Li, Ayesha Zahid, Hylemariam Mihiretie Mengist, Guy-Armel Bounda, Ying Zhou, Tengchuan Jin

**Affiliations:** ^1^Department of Obstetrics and Gynecology, The First Affiliated Hospital of University of Science and Technology of China, Division of Life Sciences and Medicine, University of Science and Technology of China, Hefei, China; ^2^Division of Molecular Medicine, Hefei National Laboratory for Physical Sciences at Microscale, CAS Key Laboratory of Innate Immunity and Chronic Disease, Division of Life Sciences and Medicine, University of Science and Technology of China, Hefei, China; ^3^Gabonese Scientific Research Consortium, Libreville, Gabon; ^4^Department of Clinical Pharmacy, School of Basic Medicine and Clinical Pharmacy, China Pharmaceutical University, Nanjing, China; ^5^Sinomedica Co., Ltd., Mong Kok, Hong Kong; ^6^Chinese Academy of Science Center for Excellence in Molecular Cell Science, Shanghai, China

**Keywords:** epidemiology, human papillomavirus (HPV), HPV-related disease, molecular pathogenesis, cervicovaginal microbiome, natural history, intratypic molecular variant

## Abstract

Diagnosed in more than 90% of cervical cancers, the fourth deadliest cancer in women, human papillomavirus (HPV) is currently the most common pathogen responsible for female cancers. Moreover, HPV infection is associated with many other diseases, including cutaneous and anogenital warts, and genital and upper aerodigestive tract cancers. The incidence and prevalence of these pathologies vary considerably depending on factors including HPV genotype, regional conditions, the study population, and the anatomical site sampled. Recently, features of the cervicovaginal microbiota are found to be associated with the incidence of HPV-related diseases, presenting a novel approach to identify high-risk women through both blood and cervical samples. Overall, the HPV repartition data show that HPV infection and related diseases are more prevalent in developing countries. Moreover, the available (2-, 4-, and 9-valent) vaccines based on virus-like particles, despite their proven effectiveness and safety, present some limitations in terms of system development cost, transport cold chain, and oncogenic HPV variants. In addition, vaccination programs face some challenges, leading to a considerable burden of HPV infection and related diseases. Therefore, even though the new (9-valent) vaccine seems promising, next-generation vaccines as well as awareness programs associated with HPV vaccination and budget reinforcements for immunization are needed.

## Introduction

Human papillomavirus (HPV) is the most common sexually transmissible infection (STI) in the world, with a high negative impact on individual social life. In their lifetime, sexually active women and men will be infected at least once (1), without necessarily developing any pathologies. Belonging to the *Papillomaviridae* family, HPV is a small, double-stranded DNA virus classified into two categories: low-risk HPVs (LR-HPVs) responsible for anogenital and cutaneous warts, and high-risk HPVs (HR-HPVs) responsible for oropharyngeal (oral, tonsil, and throat areas) cancers and anogenital cancers, including cervical, anal, vulvar, vaginal, and penile cancers (2–6). Cervical cancer (CC), the third most prevalent cancer in women (7), is an HPV-related disease for which the burden is the most blatant, as it leads to high mortality in women after breast cancer (8). Overall, the epidemiologic distribution of HPV infection and HPV-associated burden vary significantly across the world, and the morbi-mortality–associated factors include geographic, socioeconomic, cultural, and genetic factors related to viral genome variability as well as intrinsic individual factors such as age, gender, anatomic site, and health state (9). The three types of currently approved HPV vaccines, including bivalent, tetravalent, and 9-valent vaccines, are effective in reducing HPV infection and HPV-related disease incidence, as reported in several world regions. This effectiveness lies in the fact that they target and induce immunity against LR- and HR-HPVs responsible for 70 and 90% of genital and cutaneous warts and cancers, respectively. However, despite the proven efficacy of HPV vaccines, the burden of HPV-associated cancer and disease remains high.

Epidemiological surveillance of HPV infection and related diseases represents a crucial topic for monitoring and evaluation of the three currently available antiviral prophylactic vaccines (2-, 4-, and 9-valent vaccines), as well as their acceptance all over the world. Moreover, knowledge of HPV infection characteristics and evolution at the molecular level allows for a better understanding of the actual distribution of the burden of HPV-related diseases and their impact. This would in turn inform new therapeutic strategies for the development of next-generation antiviral vaccines, to overcome the shortcomings of current prophylactic regimen, including high costs, limited antiviral protection spectrum, and immunization management. In this review, we summarize the current prevalence distribution of HPV infection and related diseases worldwide and discuss the involvement of factors, specifically the genetic and molecular characteristics of the antigenic HPV-L1 protein, and reveal microbial factors associated with the natural history of HPV. We also present the actual infection progression worldwide with an emphasis on its evaluation, specifically the limitations of current HPV vaccines, highlighting the improvements to be made to reduce the burden of this disease.

## Epidemiology of HPV Infection and Related Diseases

### Epidemiology of HPV Infection Worldwide and by Region

HPV infection has reached a considerable proportion worldwide, particularly among women, in whom it is the primary cause of cancer (10), thus making HPV a current public health priority. With an estimated 291 million HPV-positive women worldwide in 2007 (11), HPV infection has remained one of the most common viral infections in the world.

Among women with normal cervical cytology (NCC), earlier reports show 10.4% (11) and 11.7% (12) HPV prevalence in 2007 and 2010, respectively, adjusted to 9.9% in 2019 (7). The highest HPV prevalence in these women was found in Oceania (21.8%, estimated to 30.9% in 2019) and Africa (21.1%), followed by Europe (14.2%), America (11.5%), and Asia (9.4%) (7, 11, 12). In the general female population, 32.1% of 576,281 gynecologically healthy and unhealthy women were HPV carriers in 2011, and Asia and Africa were found to have the highest prevalence of 45.5 and 29.6%, respectively (13). No available data were found for Oceania.

The intra-continent distribution of HPV prevalence fluctuates significantly, allowing worldwide regional repartition.

Regarding the geographical world regions (7), the HPV distribution profile in women with NCC is practically similar to that of the general female population. It is higher in developing regions. The compiled studies from cytologically healthy women showed that the HPV prevalence was higher in Sub-Saharan Africa (SSA) (24.0%), specifically in the regions of Eastern Africa (33.6%) and Latin America (7, 12). In all females, the highest prevalence was found in Asian regions, where almost half of Eastern and Central and Southern Asians (57.7 and 44.4%, respectively) were carriers, and in the SSA region, 42.2 and 32.3% of women in Southern and Eastern Africa were HPV carriers, respectively. In almost all European countries, the HPV prevalence was low (<30%), as in Western Europe (3.7%). Therefore, HPV infection rates are higher in developing regions (42.2%) than in developed regions (22.6%) (5, 13–15). Nevertheless, the prevalence is quite high in Eastern Europe (21.4%) and low in North Africa (9.2%) and Western Asia (2.2%), regardless of development (3). In addition, in all these studies involving females, similar trends were observed in terms of age. Adolescent girls and women under 25 were the most infected (3, 12, 16), although in the African (East and West Africa) and American (Central and Southern America) regions, there was a rebound in HPV infection in adults above 45 years old (3, 7, 12, 17).

In men, the global prevalence rate of genital HPV infection is almost similar to that in women (3.5–45% vs. 2–44%) (18, 19); the transmission rates being similar as well (20). This is quite understandable because anogenital HPV is mostly sexually transmitted (21). Indeed, in a recent study conducted in 2014, 9.0% of 4,065 healthy men from Africa, America, Asia, and Europe were found to be HPV carriers (22). Homosexuals and HIV-infected men are at increased risk, with higher incidence rates (≥90%) of HPV anal infection (23) than those in heterosexual men, in whom the number of sexual partners determines the risk of HPV infection. In addition, for all men, the HPV infection rate is as high among the youngest as it is among the oldest, and varies very little with age (24). This trend is different from that previously reported in women (12). In terms of geographical distribution, the incidence rate of HPV infection in men, as seen among women, is higher in Africa, especially in South African men (17.2% per year), and lower in Asia (3.2% per year) (22). Findings from a study conducted by Giuliano et al. confirmed the higher prevalence of all HPV genotypes in low- and middle-income countries compared with those in developed regions (25).

Taken together, these data lead to the conclusion that the most blatant HPV incidence and prevalence are recorded in the low- and middle-income regions in both genders, healthy and unhealthy (7). In fact, it is well-known that poverty, associated with idleness, is the main cause of transmission of sexually transmitted infections, including HPV. Furthermore, because most women, in general, start sexual intercourse early (16, 26, 27), this reasonably explains the very high prevalence rates of high-risk oncogenic genital HPVs (HR-HPVs) observed in young women, especially in low- and middle-income countries. Interestingly, in some underdeveloped regions, such as in SSA, and in East and Southwest Asia, patterns of cultural diversity (28), early marriages followed by high divorce rates, are crucial factors in increasing viral transmission (27). Moreover, in SSA, access to screening and healthcare remains a real battlefield (29), a challenge compared with developed countries, especially in women when they marry very early (10–14 years old), leading to a lack of health empowerment (27).

Due to their competent immunity, and their sexual and financial stability, some women generally eliminate the infection very quickly, explaining the decrease in HPV prevalence after 25 years of age. The low rates of persistence, recrudescence, or reinfection, observed in older women, are generally caused by the multi-sexual partnership due to social and financial instability (poverty) and lack of education (26, 30).

Second, within only 3 years (from 2007 to 2010), the overall prevalence of HPV infection in women with NCC increased by about 1% (10.4–11.7%, respectively), with the same trend as those described in the different sub-continents mentioned earlier (11, 12). These data suggest that by 2025, an increase of about 5% in cases of healthy HPV-positive women could be observed worldwide (~17%) if no current vaccination program is implemented or improved, especially in low- and middle-income countries. Because of the dynamics of transmission (21), the increasing rate will be the same in men, even though the burden is lower. It is worth noting that even if the HPV burden is low in men than women, this does not mean they are poor carriers of the virus. Indeed, Beachler et al. reported that the clearance rate of HPV is lower in men, an idea not supported by studies carried out by Wei et al. (31, 32). However, considering the large number of samples obtained from 18 countries combined (>24,000 participants) and a relatively long period of observation (1950–2015) in this study, it is reasonable to admit that men have a low clearance rate because they are less likely to develop natural anti-HPV immunity compared with women (33) who clear the infection within a relatively shorter period than men.

In men, the high prevalence of HPV, which is invariable with age, could be explained by their sexual activity preferentially with young girls under 25 who are highly infected (26), and by the fact that men compared with women have a low potential of developing natural or acquired immunity, even after repeated exposures (33). This suggests that men are reservoirs or vector-based niches for HPVs (especially HR-HPVs) for women—and MSM—in whom conversely, any sexual character is a potential transmission method. From this statement, we can speculate that the 1% increase in the infection rates observed in women could be similar in men.

### Epidemiology and Regional Burden of HPV-Related Diseases

#### Cutaneous Warts

Cutaneous warts (CWs) are mainly caused by the genera β and γ (HPV4 and 65), and rarely by the genera α (HPV2, 27, and 57) and μ (HPV1) from low-risk oncogenic genital HPVs (LR-HPV), called cutaneous HPVs (34, 35). A recent report in Dutch children showed a high cutaneous HPV rate of 92% in CW samples (36). Ubiquitous, HPVs are detected in commensal cutaneous flora of more than 50% of healthy individuals (37), particularly in children by 4 years of age (38). The appearance of CW is, therefore, the result of viral multiplication persistence but remains transient until 1–2 dozen months (34, 36, 39). This viral persistence rate and therefore the relative risk of CW occurrence is proportional to age [66% in children and 92% in adults (40)], as children are either not exposed or are less exposed than adults to viral persistence risks, including any sexual practices, smoking, use of hormonal contraceptives, and other infections (41–44).

The CW prevalence does not exceed 33% in healthy subjects and tends to be higher in males than in females (39, 40, 45). In immunocompromised people (organ transplant recipients and HIV-positive patients), the spread of cutaneous HPV on the skin is important, and the prevalence and incidence of CW are higher (up to 90%); consequently, the risk of developing non-melanoma skin cancer is increased (45). Currently, there is a lack of data on the regional epidemiology of CW.

#### Anogenital Warts (AGWs)

Mainly caused by the low prevalence (<1%, Table 1) of HPV6 and 11 (47, 48), AGWs are the most common clinical manifestations of HPV infections known worldwide (49). The morbidity associated with AGW seems low in general, as LR-HPV infections are transient. However, the cost of regular diagnosis and treatment, the stress, and declining quality of lifestyle related to this condition are important because of high transmission and frequent recurrences (5, 50–52). Considered as one of the major risk factors for the development of anal and oral cancers, AGW is found in 80–85% of anal cancers (53) and 50% of cancers of the upper aerodigestive tract (oropharyngeal cancer) (54).

**Table 1:** Distribution and prevalence of HPV genotypes by regions around the world in both women with normal cervical cytology (NCC) (12) and women with invasive cervical cancer (ICC) (46).

**HPV strains**	**World**			**Europe**		**Latin America and the Caribbean**		**North America**		**Asia**		**Africa**	
	**Mean**	**NCC**	**ICC**	**NCC**	**ICC**	**NCC**	**ICC**	**NCC**	**ICC**	**NCC**	**ICC**	**NCC**	**ICC**
HPV 6	0.3%	0.5%	0.1%	0.5%	0.1%	0.9%	<0.1%	2.0%	··	0.2%	<0.1%	··	··
HPV 11	0.1%	0.2%	<0.1%	0.3%	··	0.7%	<0.1%	··	··	0.1%	<0.1%	··	··
**HPV 16**	**32.1%**	**3.2%**	**61.0%**	**4.8%**	**65.5%**	**3.3%**	**59.2%**	**5.8%**	**71.9%**	**2.5%**	**60.5%**	**3.5%**	**47.6%**
**HPV 18**	**5.8%**	**1.4%**	**10.2%**	**0.9%**	**7.3%**	**1.2%**	**9.1%**	**2.3%**	**6.9%**	**1.4%**	**11.2%**	**1.8%**	**22.6%**
HPV 26	0.2%	<0.1%	0.3%	··	0.1%	··	0.5%	··	··	<0.1%	0.4%	··	··
HPV 30	0.2%	<0.1%	0.3%	··	0.2%	··	0.4%	··	··	<0.1%	0.3%	··	0.6%
**HPV 31**	**2.3%**	**0.8%**	**3.7%**	**2.3%**	**3.4%**	**1.2%**	**4.9%**	**1.0%**	**3.1%**	**0.3%**	**3.0%**	**1.8%**	**1.8%**
HPV 32	<0.1%	<0.1%	··	··	··	··	··	··	··	<0.1%	··	··	··
**HPV 33**	**2.2%**	**0.5%**	**3.8%**	**0.6%**	**5.7%**	**0.8%**	**3.5%**	··	**3.1%**	**0.3%**	**3.5%**	**1.1%**	**1.5%**
HPV 34	<0.1%	<0.1%	<0.1%	··	<0.1%	··	<0.1%	··	0.6%	<0.1%	<0.1%	··	··
**HPV 35**	**1.2%**	**0.5%**	**1.9%**	**0.2%**	**2.2%**	**0.7%**	**2.1%**	··	··	**0.1%**	**1.0%**	**1.8%**	**5.0%**
**HPV 39**	**1.1%**	**0.6%**	**1.6%**	**0.8%**	**1.3%**	**0.4%**	**2.2%**	**1.2%**	**1.3%**	**0.4%**	**1.2%**	**1.1%**	**0.6%**
HPV 40	<0.1%	0.1%	··	··	··	0.2%	··	··	··	0.1%	··	··	··
HPV 42	0.2%	0.4%	<0.1%	0.2%	0.1%	0.4%	··	··	··	0.3%	··	1.2%	··
HPV 43	<0.1%	0.2%	··	··	··	··	··	··	··	0.2%	··	··	··
HPV 44	0.2%	0.4%	<0.1%	0.1%	<0.1%	0.3%	··	··	··	0.3%	··	1.0%	··
**HPV 45**	**3.2%**	**0.5%**	**5.8%**	**0.4%**	**3.9%**	**0.7%**	**6.8%**	**1.0%**	**5.6%**	**0.3%**	**5.5%**	**1.7%**	**9.9%**
**HPV 51**	**1.0%**	**0.6%**	**1.3%**	**0.4%**	**1.4%**	**0.5%**	**1.6%**	**1.5%**	**1.3%**	**0.5%**	**0.7%**	··	**2.4%**
**HPV 52**	**1.9%**	**0.9%**	**2.8%**	**0.4%**	**1.9%**	**0.7%**	**2.7%**	**2.1%**	**3.1%**	**0.7%**	**3.8%**	**2.4%**	**2.6%**
**HPV 53**	**0.5%**	**0.6%**	**0.3%**	**0.3%**	**0.5%**	**0.5%**	**0.3%**	**1.2%**	**0.6%**	**0.5%**	** <0.1%**	**1.2%**	··
HPV 54	<0.1%	0.4%	··	0.1%	··	0.5%	··	1.1%	··	0.2%	··	0.9%	··
**HPV 56**	**0.7%**	**0.6%**	**0.8%**	**0.3%**	**1.6%**	**0.4%**	**0.6%**	**1.0%**	**0.6%**	**0.5%**	**0.7%**	**1.2%**	**0.7%**
HPV 57	<0.1%	<0.1%	··	··	··	··	··	··	··	<0.1%	··	··	··
**HPV 58**	**1.5%**	**0.7%**	**2.3%**	**0.4%**	**1.3%**	**1.0%**	**2.0%**	**1.5%**	**1.9%**	**0.5%**	**3.9%**	**1.6%**	**0.7%**
**HPV 59**	**5.2%**	**0.3%**	**1.1%**	**0.2%**	**0.7%**	**0.4%**	**1.2%**	**1.2%**	··	**0.2%**	**1.4%**	··	**0.2%**
HPV 61	<0.1%	0.2%	<0.1%	0.2%	··	0.5%	<0.1%	··	··	0.1%	··	··	··
HPV 62	<0.1%	0.2%	··	0.1%	··	0.4%	··	0.7%	··	0.1%	··	··	··
**HPV 66**	** <0.1%**	**0.5%**	** <0.1%**	**0.6%**	** <0.1%**	**0.4%**	** <0.1%**	**1.3%**	··	**0.2%**	** <0.1%**	**1.5%**	**0.4%**
HPV 67	0.2%	0.1%	0.3%	··	0.1%	··	0.4%	··	··	0.1%	0.4%	··	··
**HPV 68**	**0.5%**	**0.3%**	**0.7%**	**0.2%**	**0.6%**	**0.4%**	**0.6%**	··	··	**0.1%**	**0.9%**	**0.9%**	**0.2%**
HPV 69	<0.1%	0.1%	<0.1%	0.1%	··	··	0.2%	··	··	0.1%	··	··	0.2%
HPV 70	0.2%	0.3%	0.1%	0.3%	<0.1%	0.5%	<0.1%	··	··	0.3%	0.2%	··	··
HPV 71	<0.1%	<0.1%	··	··	··	0.2%	··	··	··	<0.1%	··	··	··
HPV 72	<0.1%	0.3%	··	0.1%	··	0.2%	··	··	··	0.2%	··	··	··
**HPV 73**	**3.5%**	**0.2%**	**0.5%**	**0.1%**	**0.8%**	**0.2%**	**0.4%**	··	··	** <0.1%**	**0.5%**	··	**0.2%**
HPV 74	<0.1%	0.2%	<0.1%	··	··	··	··	··	··	0.3%	<0.1%	··	··
HPV 81	<0.1%	0.4%	··	0.2%	··	0.4%	··	··	··	0.3%	··	1.2%	··
**HPV 82**	** <0.1%**	**0.1%**	** <0.1%**	··	··	··	**0.1%**	··	··	**0.1%**	** <0.1%**	··	··
HPV 83	<0.1%	0.2%	··	0.2%	··	0.4%	··	0.7%	··	<0.1%	··	··	··
HPV 84	<0.1%	0.3%	··	··	··	0.4%	··	1.0%	··	0.2%	··	··	··
HPV 86	<0.1%	<0.1%	··	··	··	··	··	··	··	<0.1%	··	··	··
HPV 89	<0.1%	<0.1%	··	··	··	0.2%	··	··	··	<0.1%	··	··	··
HPV 90	<0.1%	0.1%	··	0.1%	··	··	··	··	··	0.2%	··	··	··
HPV 91	<0.1%	<0.1%	<0.1%	··	<0.1%	··	··	··	··	<0.1%	··	··	··
Total	46.1%	7.2%	84.9%	9.7%	87.1%	17.2%	81.6%	4.8%	90.9%	10.9%	88.2%	21.3%	78.7%

*High-risk oncogenic HPV (HR-HPV) types are in bold characters*.

In children, the prevalence of AGW is low, almost zero. In contrast, several new studies revealed a net increase in the AGW prevalence in both adults and children, leading to the hypothesis of sexual abuse in children (55) because HPV has long been considered a sexually transmitted disease. However, this hypothesis is opposed by several studies which have shown that the presence of AGW in children is also associated with non-sexual HPV transmission (56, 57), particularly by manu-porterage (38) and from mother to child (57, 58).

In the general adult population, the incidence (194.5/100,000) and prevalence (0.13–0.56%) of AGW are similar in women and men, with peaks observed between 20 and 29 years of age (59). This may be due to the fact that young adults are more likely to engage in frequent sexual encounters, substance abuse, abortions, and excessive use of contraceptive pills.

Regionally, SSA appears to have slightly higher AGW incidence and prevalence rates (60), especially among people with multi-sexual partners and those who are immunocompromised (41–44). In developed regions, the incidence of AGW incidence appears low (around 1.5% in the USA) and seems to decrease because of vaccine implementation (49). Although AGW is less prevalent worldwide, it considerably affects the lifestyle of the youth, especially in underdeveloped regions.

#### HPV-Related Cancers (Anogenital and Upper Aerodigestive Tract Cancers)

Each year, around 569,847 new cases of cervical cancer (CC) are diagnosed (13.1/100,000 women). In 2018, it was responsible for 3.3% of deaths due to cancers by causing nearly 311,365 deaths, with more than 85% in developing countries (7–10, 61). This death rate is estimated to be more than 443,000 by 2030, with 90% in SAA (62, 63), as long as no sanitary measure is set up or implemented.

Since the 1980s, HPV infection (HR-HPV) has been associated with CC (5, 6). It is estimated that 85% (8,977/10,575) of invasive cervical cancers (ICCs) are associated with HPV; interestingly, more than 98% of CC deaths are attributed to HR-HPVs (Table 1) (3, 46). With an estimated 18.6/100,000 cases of CC attributable to HPV in 2018, Africa (31.5/100,000 women/year), specifically SSA (75.3/100,000 women/year), had the highest incidence rate, and Asia (10.2/100,000 women/year) the lowest (7). However, most blatant CC-associated deaths remain in Asia and Africa, particularly in low- and middle-income countries. Apart from CC, HPVs (commonly 6, 11, 16, 18, 33) are responsible for other cancers such as anogenital cancers, including anal (88%), vulvar (43%), vaginal (70%), penile (50%), and oropharyngeal cancers (37%) (2–4). Although they are rare (4), these diseases are important because they are linked with HPV.

In summary, aside from anal, vulvar, and oropharyngeal cancers, over 90% of anogenital cancers, especially CCs, are associated with HPV (2) and are more prevalent in developing countries where they are responsible for a high death rate. Altogether, these cancers are highly morbid because they are associated with treatment-related pain syndromes (4). The observation that women and MSM are more likely at risk to develop anal and oropharyngeal cancers can be explained by the fact that men, acting as reservoirs, transmit HPV to other men and women through anal intercourses, increasing the risk of HPV-related cancers. In developed countries, high alcohol consumption and cigarette smoking account for the incidence of oropharyngeal cancer. Furthermore, immunosuppressed people, and women whose partners are uncircumcised, are at higher risk due to low genital HPV clearance and high HPV prevalence in the foreskin of uncircumcised men, respectively (33, 64, 65). This indicates that in addition to the use of HPV vaccines and condoms, the circumcision of males at a young age would be an important HPV prevention strategy.

#### HPV Infection and Other Cancers (Prostate and Breast Cancers)

Unlike the evident association between HPV infection and the previously cited cancers such as CC, the real implication of HPV in the development of prostatitis is very complex because other viral and bacterial pathogens including Epstein–Barr Virus (EBV), *Chlamydia tracomatis*, and *Propionibacterium acnes* have significantly been identified in prostate cancer (PC) tissues. The viral load and HPV prevalence in the same tissues are also low (66, 67). However, several studies have demonstrated a link between HPV infection and progression of prostatitis from benign cancerous states to malignant states, as HR-HPVs, present in benign prostate tissues, have been shown to immortalize prostate cells. These studies used several biological tools on both samples (malignant and benign from the same patients), particularly PCR and RNASeq targeting L1 gene, and E6 and E7 oncogenes, to demonstrate the presence of HPV 16, 18, 45, 47, 76, and 115, suggesting that although low in viral load, the hyper-expression of HR-HPV oncoproteins (E7) in benign tissues is responsible for the development of malignant prostate cancer (66, 68).

Likewise, several studies debate the implication of HPV in the occurrence of breast cancer (BC) (69, 70). However, despite data revealing a high prevalence of HPV in cancerous tissues (69), the involvement of HPV as an etiological agent in the occurrence of BC remains inconclusive as for PC (70, 71).

In summary, the presence of HR-HPVs in an organ or tissue susceptible to a tumor (other than those with a direct link with HPV) implies that HR-HPVs would be involved in cell immortalization, and therefore in the progression to malignant states in the tissue or organ involved. Thus, the presence of HPV in mammary or prostatic tissues should not be considered a carcinogenesis etiological factor until proven otherwise but rather as a risk factor for aggravation of already established benign states toward metastatic states (66, 68, 70, 72).

Data based on HPV genotypic epidemiology and the presence of disease clusters detailed herein would reinforce the implication of HR-HPVs as an aggravating factor, but not as an etiological factor. The prevalence and incidence of PC and BC are higher in European and North American regions/populations. Yet, in developing countries, including Latin America and the Caribbean, and SSA, the death rate due to these cancers, as for CC, is the highest (8, 66, 73). This mortality rate perfectly correlates with the prevalence of African HR-HPV variants shown to be the most virulent in the world (see section HPV Genetic Variability and Prevalence Distribution of HPV Infection and Related Diseases). Moreover, the mortality rate due to these cancers is high in developed regions with high vaccination coverage rates (after Australia) and a low HPV incidence, but where the PC and BC prevalence and incidence are the highest. This excludes HPV as a necessary etiological factor for these cancers but establishes it as an aggravating risk factor.

Moreover, co-infections with other pathogens (66, 73) serve as aggravating factors for prostate cancers along with HR-HPVs in developing regions where the epidemiologic cluster is variably very high and where access to care is limited.

Therefore, death rates related to such diseases can be reduced as HPV infection is a preventable STI.

### Distribution and Prevalence of HPV Genotype

Although the HPV infection-associated diseases are the same according to viral types (HR-HPV or LR-HPV, genital or cutaneous HPV) around the world, there is nevertheless a variability of HPV genotypic prevalence by geographical region. Knowledge of the genotypic prevalence in a given continental region will facilitate policy-making for strengthening vaccination programs in these specific regions or, to a lesser extent, develop a new generation of broad-spectrum vaccines. Strengthening vaccination programs or choosing the vaccine under the influence of the most prevalent genotypes will help reduce the health and socioeconomic burdens related to HPV infection (74).

Some HPV genotypes are present all over the world. Indeed, several studies have shown that the most common types are HR-HPVs, with genotypes 16, 18, 59, 45, 31, 33, 52, 58, 35, 39, 51, 56, and 53 mainly found in descending order of prevalence. Among the LR-HPVs, the most common are HPV 6 and 11 types, which are responsible for almost all GWs. However, their prevalence differs by region (Table 1) (7, 11, 12, 46, 75).

Briefly, unlike the HPV global distribution described earlier, HPV 16 and 18, detected in 71% of ICC, are less prevalent in developing regions (Table 1) (12), where the HPV-associated burden is high. To understand this antiparallel fact, it is important to know that the occurrence risk of CIN 1/2/3 and CC also depends on the geographic variability of viral variants described hereafter. Indeed, it has been proven that African HPV 16, 18, 33, and 45 variants are more virulent than other HPV variants (76–80). Moreover, HR-HPVs are more prevalent in underdeveloped countries, mostly due to a higher percentage of immunocompromised people, shortage and/or lack of access to healthcare, and the weakness of vaccination programs. For instance, the prevalence of HPV 66, which is classified as a high-risk genotype, is slightly higher in the underdeveloped regions, particularly in Africa in the case of CC (Table 1).

Genotypes 6 and 11 are the most common LR-HPVs in America and more or less in Europe, while in Asia and Africa, they are not very common. For instance, as shown in Table 1, the prevalence and incidence of HPV 6 and 11 are low in Africa. Interestingly, several LR-HPVs such as HPV 26, 34, 61, 62, 83, and 84 found in other parts of the world have not yet been identified in Africa (Table 1) (22). Thus, as described for HR-HPVs, there would be genetic variability in LR-HPVs (mainly in HPV 6 and 11) such that low-risk African variants are the most virulent. Otherwise, the exclusive presence of some LR-HPVs in Africa (HPV 44, 70, 74 with 11% prevalence) (43) leading to HPV-associated diseases (GW) and their burdens, could be due to bad living conditions, poor hygiene, a high prevalence of certain infections (HIV, Chlamydia) that weaken the immune system (41) as well as a higher prevalence of other HR-HPVs (HPV51 and 52) than elsewhere (41, 43, 81). Northern America is the region where HPV is genotypically the least diversified, while in Asia, this diversity is greater.

Finally, there is no positive correlation between the distribution of the regional prevalence of HPV genotype and the burden of the associated diseases. Rather, this HPV-related burden is associated with molecular and genetic characteristics of HPV, revealing a regional variability within HPV, which we describe hereafter.

## Pathogenesis of HPV Infection

### Molecular Basis of Variability in HPV L1 Protein and Pathophysiology

**Figure 1:**
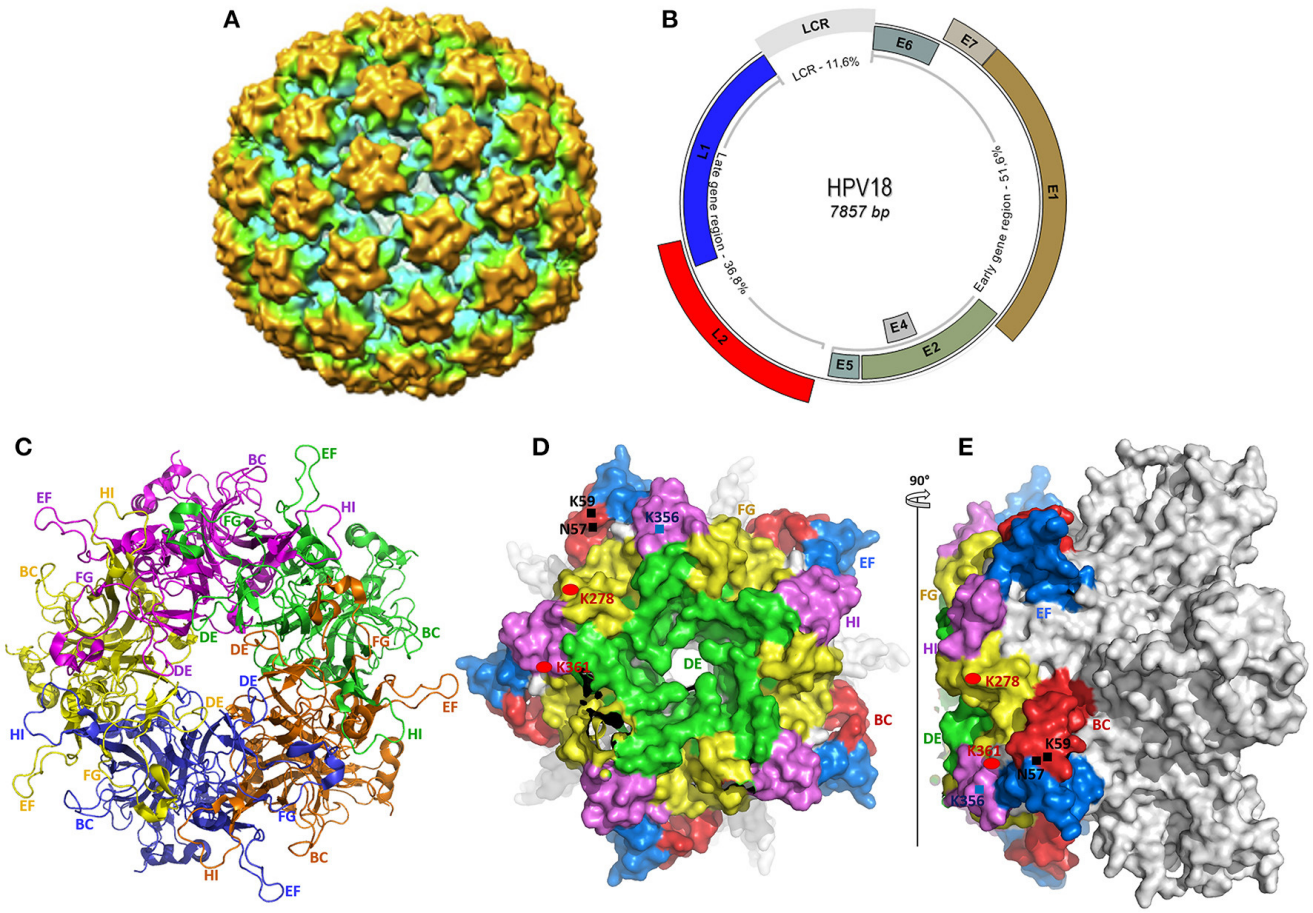
Structural representation of HPV and the L1 protein used as antigen in current vaccines. **(A)** The cryo-electron microscopy reconstitution of a whole HPV 18 viral particle, solved to a final resolution of 19 Å (82). **(B)** The viral genome representation of α-HPV (HPV18), constructed from the complete genome sequence of HPV18 (GenBank accession number NC_001357.1). All early and late ORFs, and the long control repeat (LCR) are shown in the respective proportions. **(C–E)** The crystal structure of HPV18 L1 pentamer, drawn from the PDB ID 2r5i, using PyMol (http://www.pymol.org/funding.html). **(C)** shows the top core structure of each L1 monomer in the capsomer in different colors revealing the surface loops serving as epitopes for neutralizing antibodies in VLP-based vaccines. **(D,E)** show the surface conformation in different views (top and side view), revealing each loop in different colors and some specific heparan-sulfate proteoglycan (HSPG) binding sites rich in lysine (K). Both variable and constant regions are clearly seen. The BC loop (49–66), DE loop (111–155), EF loop (169–190), FG loop (262–291), and HI loop (348–361) are clearly represented in **(C–E)**.

HPVs are 50–60 nm in diameter, non-enveloped, small double-stranded DNA viruses belonging to the *Papillomaviridae* family. The 72 capsomeres covering the virus are repetitions of pentameric monomers composed of five identical L1 proteins, anchoring one L2 protein (Figure 1A) (83). Only one strand of the 7–8 kb circular genomic DNA encodes for the eight functional early (E–E8) and two structural late (L1 and L2) proteins (84), and carries a non-coding region called the long terminal region (LTR) (Figure 1B).

The ~55 kDa major capsid protein (L1), encoded by a 1.7-kb ORF (nucleotides 5,430–7,136 from HPV18 genome in Figure 1B), consists of variable and constant regions (Figures 1D,E). The former is specific for the different HPV genotypes and constitutes the loops (BC, DE, EF, FG, and HI, Figures 1C–E) carrying the surface-specific antigenic epitopes (85) that interact with the host's membrane receptors during the cell entry process and are responsible for the production of neutralizing antibodies (86). Despite their residual variability, these loops have an identical three-dimensional structure conserved within HPVs. The latter is in the highly conserved regions within the same HPV types, with the same roles, including membrane receptor binding, L1–L1, L1–L2, and L1–L2 interactions (85, 87, 88).

This protein is the current subject of several studies on HPV therapy because of the presence of high-affinity domains with the host, responsible for stimulating the immune response (89), and specifically its ability to self-assemble into highly immunogenic, non-infectious virus-like particles (VLPs) (87, 90, 91). The immunogenic surface loops described as hypervariable regions of L1 protein play an important role in the existence of variability within HPV variants responsible for specific virulence or persistence risk for disease occurrence according to regions as described later. The intratypic variability in association with the distribution of the HPV-associated burden is discussed in the discussion section. Moreover, the naturally occurring variation within the L1 gene is also involved in the production of type-specific neutralizing monoclonal antibodies.

### HPV Genetic Variability and Prevalence Distribution of HPV Infection and Related Diseases

**Figure 2:**
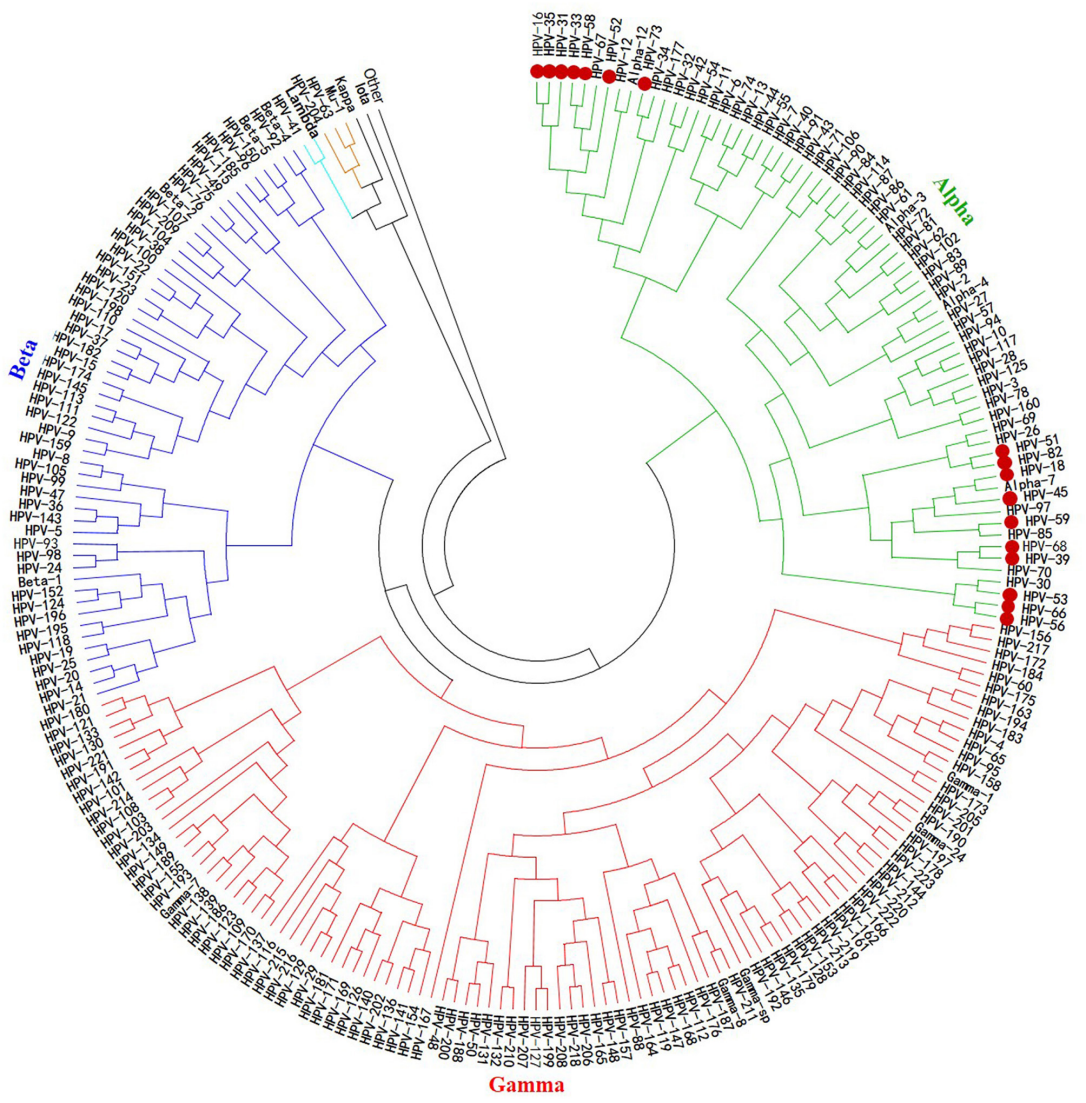
Phylogenetic tree of HPVs. The 200 HPV L1 protein sequences used to build this tree were retrieved from NCBI using the BLASTn tool, from an HPV L1 protein sequence blast. This phylogeny reconstruction was based on the neighbor-joining method with 500 bootstrap replications using MEGA7 software. It shows that all the oncogenic HPV types are clustered together within the same alpha gender. The actually considered 17 HR-HPVs are presented as bold red dots.

Although the rate of mutations causing diversity in papillomavirus is low and slowed by the corrective activity of cellular DNA polymerases during viral replication (92, 93), there is nevertheless considerable genetic variability within papillomaviruses, especially in HPVs. Until recently, more than 200 different HPV genotypes have been described and characterized, about 75% of which have been completely sequenced and published in the GenBank and EMBL databases. These genotypes belong to five phylogenetic genera, namely, alpha (α)-, beta (β)-, gamma (γ)-, Mu (μ)-, and Nu (ν)-papillomavirus (Figure 2), the only papillomavirus capable of infecting humans (94).

The classification and genetic variability within HPV are based on the sequential parsimony identity of the viral genome. Indeed, based on a pairwise comparison of the genetic sequences coding for HPV L1 protein, a new isolate is defined as belonging to the HPV type when its complete genome is sequenced and if there is <90% homology with an already known strain of HPV. Thus, two HPV strains belong to the same genus (α, β, γ, μ, or ν) when they have a homology >60%; to the same genus and the same species (α1, α2…, β1, β2…, γ1, γ2…, etc.) when they have a homology > 70%; to the same genus, the same species, and the same sub-type when they have a homology between 90 and 98%; and are different variants when they have <2% homology difference (94, 95).

Further, based on comparative genomic criteria, the existence of a geographical genotypic variability within the HPV variants has been described, referred as “inter-population or intra-type variability or intratypic molecular variants.” Indeed, comparative studies of genetic sequences of LCRs and ORFs encoding E1, E2, E5, and/or E6 proteins and, more specifically, the L1 protein showed that in the same geographical region, variants from the same HPV strain have a very low amount of divergence (high internal homogeneity), whereas this divergence between variants from two distinct regions is higher. The existence of these natural intratypic molecular variants had been demonstrated years ago from comparisons of the LCR sequences of HPV-16, proving to be specific or more widespread in certain parts of the world (96). Based on these results, HPV variants were classified into five main groups (or lineage) and further into distinguished subgroups (not detailed here): European (E), Asian (As), Asian-American (AA), African 1 (Af-1), and African 2 (Af-2). These main groups or lineages perfectly correspond to the existing main human races: Africans (Af-1 and Af-2), Caucasians (E), and Asians (As and AA) (96). This statement, based solely on studies of HPV 16 isolates, could be generalized to all papillomaviruses (80, 96–100), as a similar description was established for the inter-population variability found in HPV 18, 33, and 45 (76, 77, 101, 102). This inter-population variability made it possible to explain that HPVs from the same type induce different gene expression profiles associated with a certain disease occurrence risk, and thus to understand why in some regions the burden of HPV-associated diseases is higher than in others, for the same variants, as presented previously (100). For instance, it has been reported that the African variants of HPVs (especially HR-HPVs) are the most virulent (80), explaining why HR-HPVs like 31 and 58 (more specifically HPV 16) are highly associated with infection progression and oncogenicity (103, 104), and despite being less prevalent in underdeveloped countries like in Africa (Table 1), are associated with higher morbi-mortality (warts, CIN2/3, cancers, and death). Therefore, whether the currently available vaccines are (in terms of immunogenicity) equally effective on all existing HPV intratypic molecular variants is uncertain, particularly on the prevalent variants in low- and middle-income countries. From the available data, the existence of intratypic molecular variants from L1 proteins, specifically from the surface loop sequences, could now explain in part why VLP-based HPV vaccines have variable efficacy (or efficiency) across the world. In other words, the high prevalence of HPV-associated burden observed in some parts of the world might be due to the low efficacy of the current VLP-based vaccines.

### Molecular Modalities of the HPV Infection Cycle

**Figure 3:**
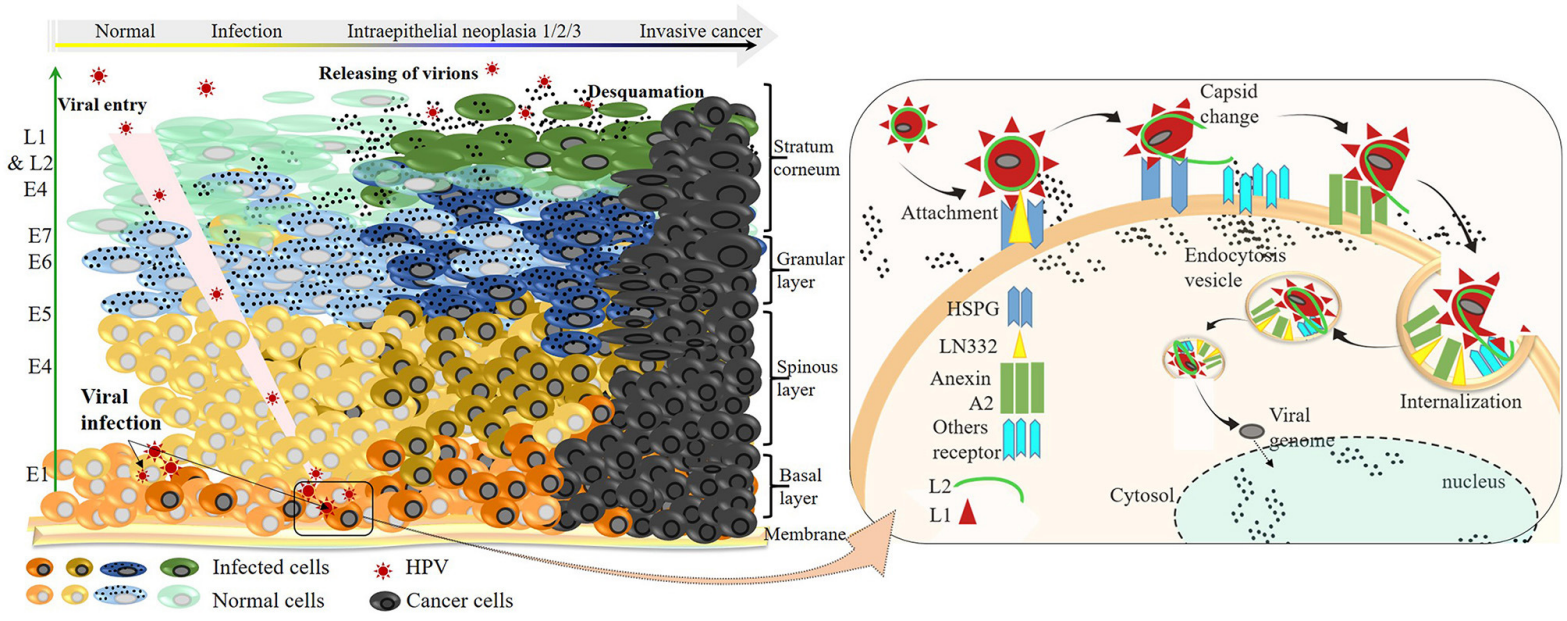
HR-HPV infection cycle at the molecular level and progression to invasive cancer. Left: HPVs enter either through a microlesion on the upper layer or directly through the squamocolumnar junction, infecting basal keratinocyte cells. Sequential viral protein expression is shown (green arrow) according to the order of their synthesis throughout the productive infection. Once infected, cells can progress to a worse infection state characterized by viral DNA integration into genomic DNA and overexpression of E6 and E7 oncoproteins. The progression of infection to the development of invasive cancer, which takes place within a long period, depends on the intrinsic factors of the infected individual and/or extrinsic of the viral strain. Right: virus–receptor interactions from the attachment to the internalization on the epithelial cells during one infection cell cycle.

Most studies describing the HPV infection cycle were conducted on a few viral strains, including HPV 16, 18, and 31 (105). However, despite the genetic variability and the related-diversified infection profile, the infection mechanism remains similar (Figure 3). HPVs infect only the undifferentiated deeper layer cells of the skin and/or mucous membranes called basal epithelial cells, which have a high mitotic capacity. Indeed, viruses present on the outer apical surface of the skin and/or mucous membranes can reach their target cells only through microlesions of these upper layer cells occurring during trauma. In rare cases, particularly in the uterine mucosa, the virus can reach the targets directly through the transformation zone between the squamous epithelium of the ectocervix and the glandular epithelium of the endocervix without mucosal tissue damage (Figure 3) (106).

The virus entry into undifferentiated epithelial cells depends on specific molecular interactions involving the viral antigens and host receptors. In fact, structural studies of L1 revealed the presence of four heparan-sulfate proteoglycan (HSPG)–specific binding sites, rich in lysine (K) (Figures 1D,E) required for productive infection (86, 107). Once in the intraepithelial environment, the first binding between the L1 and the HSPG is established. Interestingly, through its K278–K361 apolar site located on the FG and HI surface loops of two adjacent pentamers, L1 proteins bind to HSPGs; non-specific binding occurs as well with non-HSPG receptors [Laminin 332 (LN332)]. These bindings lead to the first cyclophilin B (CyPB)–dependent L1 protein 3D conformational change (108), releasing the N terminus end of L2 protein, which becomes exposed and vulnerable to the proteolytic action of furin, a cellular protease also known as protein convertase (109). The proteolysis of L2 is followed by two subsequent interactions of L1 with HSPGs, involving the K54–K356 and N57–K59/K442–K443 sites, respectively, located on FG/HI/BC and BC/α4 surface loops of two pentamers, allowing further 3D conformational rearrangements of L1 and L2, which have two important consequences for downstream infection: 1) loss of affinity of bonds involved in the first interaction, reinforcing the bonds on site 4 (K442–K443 of α4) and promoting viral movement to the free keratinocyte surface, and 2) exposure of other viral residues to a second receptor in the endocytotic compartment initiating viral internalization. Through endocytosis, the virus is transported within the small vesicles to the nucleus through the ER and the Golgi, where a series of interactions and structural changes of the vesicles allow decapsidation and release of the viral genome near the nuclear membrane. The episomal viral genome enters the nucleus through nuclear pores to initiate viral replication (110, 111).

HPV replication depends on epithelial cell differentiation. Indeed, by infecting undifferentiated basal cells, the virus guarantees its multiplication and persistence. Consistently, by dedifferentiating, the basal cells at the same time ensure viral protein synthesis sequentially, as presented in Figure 3, thus ensuring viral multiplication (latent or active) and increasing the risk of HPV infection and related diseases beforehand.

Therefore, the L1 protein is the most important in the infection process (viral entry). Owing to the presence of multiple surface epitopes, it possesses the ability to boost immunity against HPV by producing a high amount of specific and effective antibodies that recognize HPV in the physiological medium. Although the modalities of HPV infection are the same in general, as seen previously, the pattern of gene expression remains different, with differences in virulence (80) due to variability between variants based on L1 protein. Thus, a good initiative in the process of eradicating HPV infection would be to develop vaccines specifically from L1 proteins for each region, given the divergence.

### Pathophysiology, Evolution, and Natural History of HPV Infection: Risk for Disease Occurrence

In a normal cell cycle, basal epithelial cells divide asymmetrically to renew the basal layer on the one hand and the cutaneous or mucosal epithelium on the other hand. Thus, the number of divisions during cell differentiation remains limited to the formation of the apical epithelium. In contrast, HR-HPV infection leads to the maintenance of this cell division, which is the long-term origin of the HPV-related diseases presented previously. At the molecular level, it is the hyper-expression of the E6 and E7 proteins, which leads to the maintenance of cell division. Indeed, studies have shown that the overexpression of E6 and E7 oncoproteins occurs as a result of the viral genome integration into the host genome, causing a loss of expression of some viral genome parts, including E2, that negatively regulates p97 (112–114). In the absence of E2 repression activity, p97 freely induces the expression of E6 and E7, which has compromising molecular implications (115), leading to increased risk of diseases, notably HPV-related cancers (Figure 3) (112, 116, 117).

**Figure 4:**
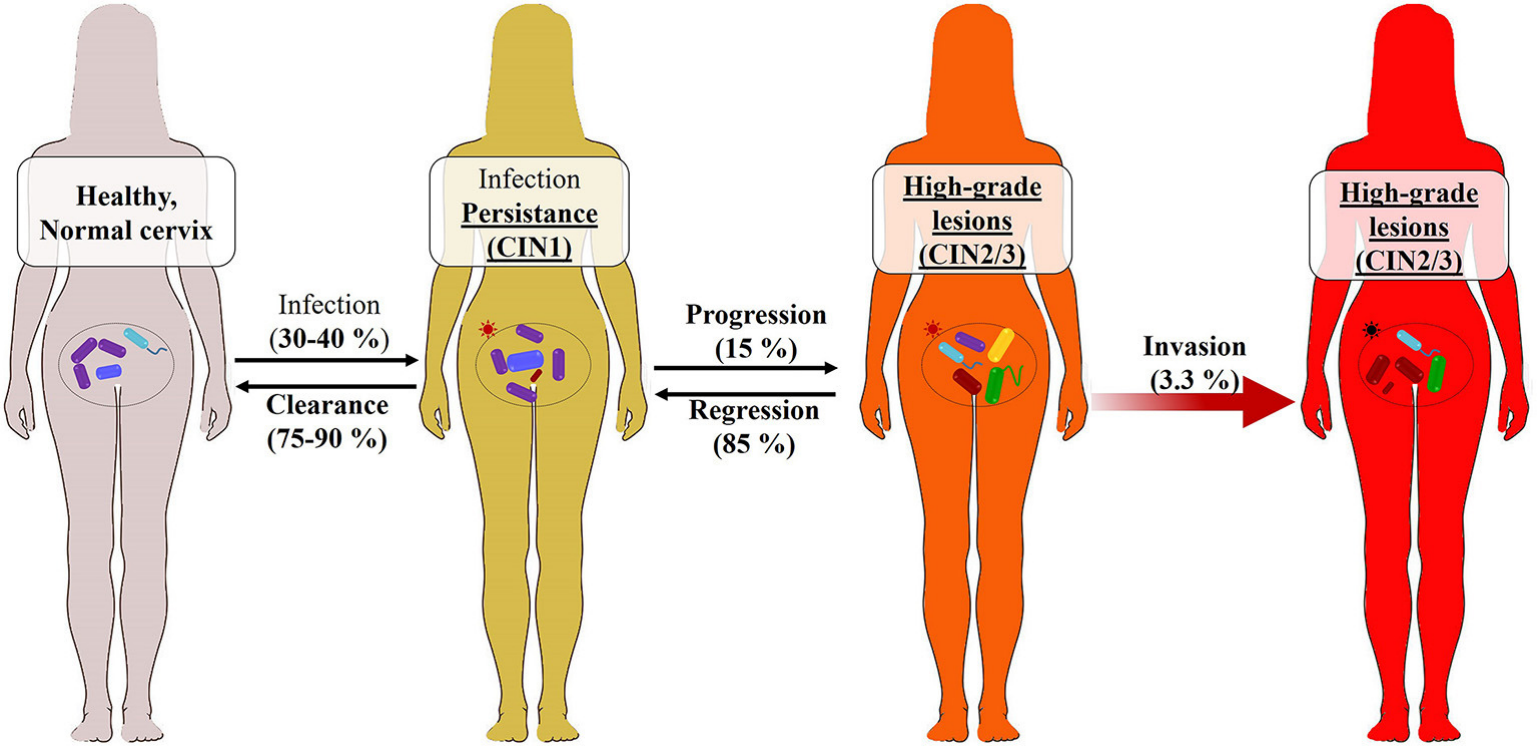
Cervicovaginal microbiome (CVM) features in HR-HPV infection natural history. This figure is an adaptation of the recently published study findings (123). The main characteristic of the CVM is its microbial diversity (*Lactobacillus* spp.—in purple, *Vibrio* and flagelled bacteria—in blue and green). Once an HR-HPV infection (red star) occurs in the 30–40% of exposed women (90%), few of them develop HPV-associated abnormalities such as low-grade cervical intraepithelial neoplasia (CIN1) or lesions, which can progress to high-grade lesion (CIN2-3) responsible in long term to invasive cervical cancers. In this figure, and as previously found, the abundance of *Lactobacilus* spp. is associated with a regression of viral persistence and a clearance of the infection. However, the progression of the infection to the precancerous lesions is associated with a subsequent increase in the microbiota variability, specifically *Gardnerella vaginalis* (in red) bacteria and some pathogenic fungi. The CVM may then serve to identify HPV-infected women with pre-cancer risk. The effect of *Gardnerella* on the CVM stability is involved in the HPV infection natural history.

Clinically, after a primary infection, the manifestations of infection are not perceptible early; this makes acute HPV infection very poorly documented. The anti-HPV antibody kinetics have not yet been determined because the prodromes associated with HPV acute infection occur very late after infection; however, studies are underway (118). Moreover, from available data, about 98% of women are exposed to HPV infection, and about 30% (119) to 40% (120) are susceptible to infection. Fortunately, HPV infection is transient in approximately 75–90% of cases in both genders. The GW, CW, and low-grade cervical intraepithelial neoplasia (CIN1) in women and penile intraepithelial neoplasia (PIN1) in men characterize this infection in the long term. The duration of virus elimination (2.5 years) depends on the HPV type or viral load, immune competencies, and anatomical site of infection. Women who experience viral clearance and have normal cervical cytology are at low risk (1.5%) of developing CIN (120–122). In the case of viral latency, viral reactivation leads to the persistence of infection characterized by the presence of CIN-PIN2/3, which can very rarely evolve (<10% exactly 3.3% of CIN/PIN2/3) to the development of ICC, as earlier presented (Figures 3, 4). Persistence of HR-HPV infection, abnormal cytology, intercourse, and immune weakness have been reported to be some of the key factors in the development of ICC. It should be noted that the persistence of the infection must be differentiated from reinfection after clearance, which presents a risk of developing CIN3 (3.4%) six times lower than the persistent infection (121, 122, 124) because of acquired immunity. Whether this statement concerns reinfections with the same HPV strain as the initial infection or with different strains is unknown. However, we can hypothesize that because of the capsid proteins' ability to trigger the production of cross-reactive antibodies, as shown in VLP studies, the risk of developing CIN2/3 when reinfected is lower than that after persistence following a *primo-infection*.

After the establishment of infection, replication is maintained by the E6 and E7 proteins, and HPV L1 protein plays only a minor role limited to the repeated binding with the neighboring cells. The modification of the natural history in terms of the infection rate reduction would be achieved by reducing the infection spread over other cells or by focusing mainly on the naïve population (children), who are not or LR-HPV carriers.

### Cervicovaginal Microbiome and Precancerous Lesion Occurrence Risks

Beyond the HPV genotypes and/or variants, sexual practices, smoking, use of hormonal contraceptives, and several viral (like HIV) and bacterial (like *Chlamydia*) infections have been associated with HPV infection progression. Furthermore, it has been revealed that a significant correlation exists between the vaginal and cervix microbiota and the natural history of HPV infection (Figure 4).

The vaginal microbiota consists of a variable bacterial population whose quantitative and qualitative changes have been associated with several gynecological disorders (125). However, its pathophysiological role in the context of HPV infection was unknown until recently. Recent studies have suggested that the cervicovaginal microbiome (CVM) plays an important role in the persistence of HR-HPVs and the occurrence risks of CIN2/3 and invasive cancers by influencing the natural history of HPV infection, and could serve as a complementary therapeutic target to prevent the occurrence of high-grade intraepithelial lesions leading to invasive cancers (126–130).

Usyk et al. (123) recently showed that *Lactobacillus iners* in CVM is a biological marker of HPV infection clearance. In other words, they demonstrated the hypothesis raised earlier (130) that increased bacterial diversity combined with depletion of *Lactobacillus* spp. in CVM is associated with the persistence of HPV infection and a higher risk of neoplastic intraepithelial lesions caused by HR-HPVs, leading to the development of invasive cancers. In addition, they found that *Gardnerella*-type bacteria (*Gardnerella vaginalis*), along with *Mobiluncus* sp., *Prevotella* sp., *Mycoplasma hominis*, and *Atopobium vaginae*, marked the persistence of HR-HPV infections and precancerous lesions (123, 130, 131). Specifically, the pathological effect of *Gardnerella* on the progression of lesions was positively mediated by an increase in microbial diversity. Therefore, by monitoring the presence of *Gardnerella* in HPV-infected women with intraepithelial lesions and high bacterial diversity in CVM, it is possible to predict a possible risk of developing invasive cancer. On the other hand, HPV-infected women with low-grade intraepithelial lesions and high abundance of *Lactobacillus* spp. are at low risk of developing cancer. This novel association between *Gardnerella* and the disruption of CVM stability influences the natural history of HPV infection.

## HPV Preventive Strategies: Challenges

So far, it is clear that vaccination is the first line of defense against HPV infection and related diseases. Several studies aimed at reducing the virus-related disease burden led to the development of three currently available vaccines and several candidate vaccines at advanced clinical trial phases (90, 132–135). These vaccines are Cervarix, Gardasil, and Gardasil9. Briefly, the first is a bivalent vaccine (2vHPV) produced by GSK (GlaxoSmithKline Biologicals SA) that targets HPV 16 and 18, the leading HR-HPVs responsible for 70% of CC (Table 1). The second is a quadrivalent product (4vHPV) from Merck (Merck & Co., Inc.), targeting HPV 6 and 11 (responsible for 90% of GW) in addition to HPV 16 and HPV 18. The last one, a 9-valent vaccine (9vHPV) from Merck, broadens the protection spectrum of the two previous ones by additionally targeting HPV 31, 33, 45, 52, and 58, the HR-HPV types responsible for about 18% of ICC (Table 1). Gardasil, Cervarix, and Gardasil9 are highly effective vaccines approved in 2006, 2009, and 2014, respectively, by the Food and Drug Administration (136, 137) to prevent HPV infections and related diseases (132, 133, 138, 139). Indeed, in Australia and Luxembourg, a significant reduction in the burden of HPV infections and related diseases was observed (140, 141) after the introduction of 2vHPV and/or 4vHPV in vaccination programs. Recently, Rossi et al. (142) reported that the three vaccines, particularly the 9vHPV, offer extensive and effective protection against AGW, CIN-PIN2/3, and some cancers. In the USA, a surveillance study showed a considerable decline in AGW prevalence, emphasizing a beneficial effect of HPV vaccination (49). Importantly, according to WHO, none of these studies revealed any major adverse effects (143). Thus, according to the prevalence and distribution of HPV across different regions of the world (Table 1), we can safely speculate that Gardasil9 alone could prevent up to 89.6% of ICCs, specifically up to 86.7% in Africa, 88.2% in Latin America and the Caribbean, 95.6% in Northern America, 91.4% in Asia, 89% in Europe and more than 87.1% in Oceania, and 90% GW worldwide. However, the question of what effect/risk the Gardasil9 dose addition has in patients who have already received one (or two) dose(s) of one of the previous vaccines arises.

All these vaccines are developed from HPV L1 proteins obtained into VLPs (90, 144) and therefore retain all the structural and immunogenic characteristics of the virion described hereinbefore (Figure 1). Because HPV vaccines act by inhibiting viral entry into cells, they become less effective when an infection is already established, and offer limited protection against reinfection or self-infection. Thus, in some countries, their acceptance and introduction into immunization programs have been followed by a waning of infection rate, like in Australia, where vaccination coverage is the highest worldwide (140). Similarly, in regions where 9vHPV is already accepted, a significant waning of HPV-related burden is expected because recently, the vaccination age group has been widened for both sexes from under 9 to almost 50 years old (121). However, the cost of vaccination remains a real obstacle, particularly in low- and middle-income countries, justifying the huge burden of HPV infection.

Molecular screening tests for HR-HPV and the screening of precancerous epithelial lesions and ICCs, which are the second line of defense, play an important role in HPV control (121). In short, the *National Screening Committee* suggests adopting the primary HPV screening test in the cervix, in association with a genotyping test rather than cervical cancer screening because the specificity (Sp) and the number of colposcopies performed after a primary HPV screening (Sp = 76.1%; *n* = 3769) were greater than those achieved after a cervical cytology screening (Sp = 47.1%; *n* = 1934) (121, 138). In addition, the adoption of primary screening would be time- and cost-effective for developing countries (145) as the primary test can also be performed from self-sampled urines with 100% sensitivity compared with the test on the cervical smear (146, 147). However, knowing that HPV can also cause penile and anal cancers, it is questionable whether HPV testing of cervix, urine, or feces is appropriate to predict the occurrence of penile and anal cancers in men and women.

Furthermore, as stated in section Cervicovaginal Microbiome and Precancerous Lesion Occurrence RISKS, it is now known that the CVM is implicated in the natural history of HPV infection, and the monitoring of bacterial variation in the CVM is now a novel and effective measure in the follow-up diagnosis of HPV-infected women and could help to identify women at risk of developing cancer. However, the causality effect needs to be determined to know whether the disruption of CVM is caused by HPV infection or the presence of a disrupted CVM is a risk factor for HPV infection and related complications. This knowledge could help in the prevention of HPV infection and related diseases.

Despite all these control strategies, particularly vaccination, there are still high rates of morbidity due to HPV and related diseases in certain regions of the world because of challenges encountered. The main challenges are two-fold: (1) immunization coverage is not complete, or the programs are not well established/followed; (2) vaccinations are usually given very late—or not—accepted. Regarding the first case, in certain regions, vaccine coverage assessment studies showed that the vaccination programs are not adequately completed in many patients who received at least one (1) dose (148, 149), mainly because of poor follow-up and high vaccine costs. To deal with this, it is recommended that governments should provide almost-free or half-cost vaccination programs, as it can increase vaccination coverage (150, 151). In addition, studies on the impact of vaccination programs in Australia and Scotland showed that two-dose programs or better one-dose programs might be adopted as they are less expensive and seem more effective than the three-dose programs (140, 152, 153). However, the effective duration of immunization and the ability to prevent the occurrence of HPV-associated cancers remain an important issue. Moreover, vaccine development systems (insect cells and yeast) are expensive; thus, next-generation vaccines based on cost-effective systems (154) are needed.

In many countries, ignorance of the susceptibility to HPV infection and associated disorders, and the lack of confidence in vaccination programs are some of the major challenges leading to a refusal of parents to vaccinate their children (151, 155). Sensitization modules in immunization programs could greatly improve immunization coverage rates in areas where vaccination programs have already been instituted (156). This lack of confidence in the HPV-vaccine explains their refusal in certain countries, such as China. In fact, concerning the probable side effects in the Chinese population, as no clinical trial had reported the same vaccine safety profile as observed elsewhere, the Chinese government delayed their approval. In 2016, these vaccines were approved (157), demonstrating their effectiveness and safety (135, 158, 159). However, vaccination coverage remained low, and the HPV-associated burden was still high in this area. Current CC prevention strategies will only allow China to achieve WHO's objectives (incidence <4 cases/100,000 women/year) if the current budget strategies are optimized and if the budget allocated for vaccination against HPV and CC screening is heightened; otherwise, an increase in the infection's burden is inevitable (160).

Finally, it is worthy of note that the high burden of HPV is also associated with the antigenic protection limits of current vaccines. Among the 17 HR-HPVs (16, 18, 31, 33, 35, 39, 45, 51, 52, 53, 56, 58, 59, 66, 68, 73, 82), these vaccines can only protect against 7 (16, 18, 31, 33, 45, 52, and 58), offering 89.6% protection (Table 1). Furthermore, the current vaccines contain Caucasian HPV genotypes and are very effective in these populations. Therefore, further broad-spectrum and specific vaccines are required. Precisely, the major preventive strategies against HPV and related diseases, including vaccination and diagnosis, are the same worldwide. These strategies would be effective only if the molecular and genetic characteristics of HPV, already revealed and well-described here, were the same all over the world. For instance, as discussed here, the HPV infection profile and related burden are strongly associated with the regional variability in HPV genetic features, which therefore explains the prevalence distribution. Taken together, the preventive strategies should be redirected to the specific regional features of HPV L1 protein used in developing vaccines.

## Discussion

While HPV is widely spread, the related infection and its incidence as well as the burden of the related diseases differ considerably by and within regions according to several factors, including both extrinsic (geographical region, socioeconomic development, culture, and molecular variability of viral genome) and intrinsic (lifestyle, age, gender, affected anatomic site, and health state) factors (9). HPV is ranked as the second most common pathogen leading to gynecological disorders and the first to cause female cancers. CC, which is the second most common cancer after breast cancer and the third most prevalent cancer in women (3, 7), is the most common disease resulting from infection with the 17 known HR-HPVs (16, 18, 31, 33, 35, 39, 45, 51, 52, 53, 56, 58, 59, 66, 68, 73, 82), followed by anal, vulvar, penile, vaginal, and upper aerodigestive cancers. Other HPV-associated diseases include anogenital and cutaneous warts. HPV implication in PC and BC, although inconclusive, shows its participation in cell immortalization, which is responsible for cancers. Overall, the burden of these HPV infections and related diseases is more important in the low- and middle-income regions/countries in both genders (7). This repartition pattern of HPV infection worldwide has increased by 1% within 3 years, and is estimated to reach 5% by 2025, while the prevalence of HPV-related diseases (CC) is expected to increase by 2% in 2030 (3). With the dynamics of transmission (21), the consequences in men living in these countries would be similar to populations in developed countries.

Like other sexually transmissible infectious pathogens (161), the risk factors favoring the spread of HPV and associated diseases (cancers, anogenital, and cutaneous warts) are more common in developing countries/regions, where the average financial income per capita is low (3). Regions such as Eastern and Central-Southern Asia, and SSA, with nearly 50% cases of infection and with the highest HPV-related burdens in the world, are specifically the regions where more than 50% of the population earn around <5 dollars (USD) a day, that is, with a very low HDI (HDI ≤ 0.555) (http://hdr.undp.org/en/content/table-1-human-development-index-and-its-components-1). Most of these countries are characterized by poor quality of life, particularly with limited access to basic needs such as healthy food, drinking water, decent housing, good hygiene, and medical care (29). Due to extreme poverty, even the young women engage in unsafe sexual practices such as prostitution to satisfy their basic needs (16, 26, 27), consequently increasing the risk of infection. Low education and/or schooling levels (26, 30) and the cultural model that requires women to marry early (27, 28) are other factors associated with a high spread of HPV and a high disease rate in young women. The young women, thus very little educated, are used unconsciously as a currency to feed the poor family. Overall, this first explains why the HPV-related burden is higher in these regions.

Furthermore, the high prevalence of other viral and bacterial diseases (epidemiologic cluster) increases the burden associated with HPV infection and HPV-associated diseases in developing countries. More interestingly, it has recently been shown that the CVM plays a very important role in the progression of infection toward the development of precancerous lesions, and therefore in the natural history of HPV infection. High variability in the cervicovaginal microbial population associated with depletion of *Lactobacillus* spp., and an increased abundance of *G. vaginalis* along with *Mobiluncus* sp., *Prevotella* sp., *M. hominis*, and *A. vaginae*, has been characterized as a marker of HR-HPV infection persistence and precancerous lesions (123, 130, 131). It is noteworthy that limited access to healthy living conditions, potable water, good hygiene, and mainly medical care associated with the high prevalence of other gynecological infectious diseases in most of these regions, especially in Africa (29), lead to high variability in the CVM, particularly associated with the development of vaginal dysbiosis and bacterial vaginosis. These vaginal affections, defined as a highly diverse CVM containing a small amount of *Lactobacillus* spp., are very prevalent in underdeveloped countries (162), thus justifying this high burden.

From an HPV molecular genetics perspective, this uneven distribution of HPV-associated burden across the world is a rigorous and rational explanation. The major HPV viral capsid proteins (L1 protein) are the most important in the early stages of infection and the reinfection process during viral persistence, and are therefore mainly responsible for the activation of the immune response (Figure 3). They are used as vaccine antigens to prevent HPV infections and the occurrence of associated diseases. These are the hypervariable antigenic loops present on the surface of the HPV capsid, which are responsible for virus–host interactions and are involved in the production of type-specific antibodies (Figures 1, 3). Moreover, studies have shown that in addition to the genetic variability present within the primary sequence of the L1 proteins, allowing an HPV type–based classification (α-HPV 16, 18, 45, etc.) (Figure 2) (94, 95), there is a non-negligible variability within variants of identical HPV types but from distinct regions (80, 96, 96–100), which can be observed in the same geographic region due to human migration (163, 164).

This variability within HPV variants called “inter-population or intra-type variability or intratypic molecular variants” described earlier has been characterized particularly from the L1 protein, especially within the hypervariable surface loops (BC, DE, EF, FG, and HI) containing the antigenic epitopes responsible for the production of antibodies (Figures 1C–E) (104, 164). This existing polymorphism within variants of the HPV L1 genes plays an important role in type-specific recognition and neutralization, in the associated virulence or oncogenicity, and in the structure of the viral capsid. In their study, Gurgel et al. (164) characterized the existence of intratypic molecular variants (lineages A, B, C, and D corresponding to the intratypic molecular variants E, Af-1, Af-2, and AA, respectively) (104) from the alignment of the L1 molecules of HPV 16, 31, and 58. They showed that the molecular changes that differentiate one intratypic molecular variant from another appear within the surface loops DE, EF, FG, and HI, involved in specific binding to MHC-I/II and T and B cells. In particular, 23 nucleotides in the HPV16-L1 gene, 9 nucleotides in the HPV31-L1 gene, and 35 nucleotides in the HPV58-L1 gene vary from one variant to another. This huge variation has an impact on oncogenic potential. Other studies have revealed that the effect of this polymorphism within variants of the L1 genes is associated with a risk of persistence of the infection and the occurrence of diseases (CIN2/3, cancers). For instance, the non-European HPV16 variants (Af-1, Af-2, and AA) are more oncogenic than European variants (E); the HPV31 variants Af-1 are more involved in persistence toward CIN3, and HPV52 Af-2 variants are seven to eight times more oncogenic than HPV52 Af-1 variants (103, 104). It appears that this L1 polymorphism occurs within the molecular bases involved in the assembly of the capsid in the form of VLPs (polymorphism in h4, β, and J regions) (164), raising the hypothesis that the 3D structure VLPs could also vary depending on the geographic variant, and therefore introduce significant variability in the oncogenicity associated with the capsid structure. In addition to studies based on L1 genes and their roles, this effect has also been demonstrated in the LCR genes and some early proteins (E) involved in viral functions (76, 77, 80, 96–102). Taken together, this polymorphic variability and the associated oncogenic potential positively correlate with the distribution data of HPV-associated disease burden and thus explains why the HPV-related burden is higher in developing regions. Specifically, the countries/regions where the burden of HPV-associated diseases is blatant are the developing countries, and the distribution of intratypic variants is such that the most prevalent variants in these regions are the Af-1 and Af-2, and AA, known as non-European variants, shown to have a higher oncogenic potential compared with the European intratypic variants found in most developed countries.

The current preventive strategies, which include diagnostics and vaccination programs with the current HPV vaccines (2-, 4-, and 9-valent vaccines) and, in particular, the difficulties linked to their implementation in certain regions, is another aspect that explains the uneven distribution of HPV-associated diseases, specifically found to be higher in less developed regions. Developed regions, including Australia, Europe, and America, where at least two of the three current HPV vaccines have been introduced, have reported a decrease in the incidence of HPV-related HPV infections, along with an absence of adverse effects (49, 121, 140–143). The success of the vaccination program lies in the fact that in these developed countries, the HPV-related disease diagnosis rate has increased, access to health care is easier, vaccination programs are well-followed until completion, and the L1 protein used in the vaccines is from Caucasian intratypic variants. However, in other regions, including China, SSA, and Latin America and the Caribbean, vaccination program implementation faces some limitations, partly accounting for the observed high morbidity due to HPV infection and HPV infection–associated diseases. Limitations of current HPV vaccines include refusal to vaccinate and/or late implementation of the vaccine program, lack or poor follow-up due to the high cost of vaccine doses, poor access to healthcare, and limited protection against some HR-HPVs (only those taken into account in the vaccine). Given that the current vaccines are developed from non-African variants, it could be speculated that these vaccines have a limited effect on the African intratypic molecular variants, positively correlating with the high HPV-related burdens. The lower efficacy of current vaccines on non-Caucasian variants should be verified, the phenomenon of “immune pressure” exerted by weakly specific immunogenicity of current vaccines should be taken into account, because this may result, by selective pressure, in the development of resistance by non-Caucasian variants against these vaccines, further compromising their effectiveness and complicating vaccination programs. Additional studies highlighting this hypothesis would make it possible to reconsider the specificity of variants in the development of new vaccines.

## Conclusion and Suggestions

HPV is a common sexually transmitted infection worldwide, which disrupts normal social life and has lethal consequences. Apart from a few exceptions, the burden of HPV infection and related diseases remains high in developing countries, and factors that explain these high rates include poor living conditions, co-infections with other pathogens, poor healthcare facilities, and high cost of vaccines. The high CVM variability caused by multiple genital infections, associated with high abundance of *G. vaginalis* and depletion of *Lactobacillus* spp., is involved in the occurrence of CINs; thus, it is a risk factor supporting the high HPV burden in developing countries. Moreover, the limited action of the current vaccine against the African intratypic variants prevalent in these regions explains the observed HPV burden because the current vaccines are not developed from these specific L1 variants, but general (or Caucasians) strains.

Therefore, even though the current vaccines have demonstrated satisfactory efficacy and safety, and even if the future expectations for the 9-valent vaccine seem satisfactory, the need to develop broad-spectrum vaccines targeting other HR-HPV variants and next-generation vaccines targeting other HPV proteins remains existential. Furthermore, the adoption of one- or two-dose vaccination programs is highly desirable, as it will reduce the cost and allow broad coverage (140, 152, 153). Awareness programs and the budget strengthened toward immunization would be beneficial in limiting recrudescence in women and lowering the burden of infections in so-called reservoirs (men) and women. In addition, the bacterial DNA test in the cervix or blood, and the monitoring of variability in the CVM in terms of HPV screening in the cervix and assessment of cervicovaginal lesions, are novel and effective measures to be adopted in the follow-up diagnosis of HPV-infected women. This could help to find a treatment method involving CVM to prevent infection progression.

## Author Contributions

AK conceived the presented idea, extracted the data, wrote the original draft, and formatted the article for submission. BL, HM, and AZ reviewed and edited the original version of the article. G-AB extensively reviewed, edited, and formatted the article for submission. YZ provided resources and technical assistance in data extraction, critically reviewed, and edited the article. TJ conceptualized the main idea, provided resources in data extraction and financial assistance during the whole study, and supervised the whole paper. All the authors read and approved the final version of the article for publication.

## Conflict of Interest

G-AB was employed by the company Sinomedica Co., Ltd. The remaining authors declare that the research was conducted in the absence of any commercial or financial relationships that could be construed as a potential conflict of interest.
